# New Insights into Tumor-Infiltrating B Lymphocytes in Breast Cancer: Clinical Impacts and Regulatory Mechanisms

**DOI:** 10.3389/fimmu.2018.00470

**Published:** 2018-03-08

**Authors:** Meng Shen, Jian Wang, Xiubao Ren

**Affiliations:** ^1^Department of Immunology, Tianjin Medical University Cancer Institute and Hospital, Tianjin, China; ^2^National Clinical Research Center for Cancer, Tianjin, China; ^3^Key Laboratory of Cancer Prevention and Therapy, Tianjin, China; ^4^Tianjin’s Clinical Research Center for Cancer, Tianjin, China; ^5^Key Laboratory of Cancer Immunology and Biotherapy, Tianjin, China; ^6^Department of Biotherapy, Tianjin Medical University Cancer Institute and Hospital, Tianjin, China

**Keywords:** tumor-infiltrating B lymphocytes, breast cancer, prognosis, regulatory mechanism, checkpoint molecules

## Abstract

Currently, tumor-infiltrating B lymphocytes have been recognized as a new hallmark of breast cancer (BC). The function seems to be controversial, either with positive, negative, or no significance in BC’s prediction and prognosis. Moreover, B-cell infiltrates regulate tumor process through productions of antibodies and interleukin-10. The interactions with other lymphocytes and programmed death-1/PD-1 ligand axis are also documented. The regulatory mechanisms will eventually be incorporated into diagnostic and therapeutic algorithms, thus give guide to clinical treatment. In this review, we give new insights into clinical impacts and regulatory mechanisms of tumor-infiltrating B cells, which heralds a new era in immuno-oncology in BC treatment.

## Introduction

Breast cancer (BC) is a major worldwide health burden, which is linked with increased prevalence of malignancy-related mortality mostly among females (1). Current evidence demonstrated that the intensity of tumor immune response may influence the effectiveness of BC therapy, and probably affect clinical outcomes. However, the roles of tumor-infiltrating B cells (TIL-B) and plasma cells (PC) in cancer therapy still merit future investigations. Neither histopathological features nor conventional biomarkers are accurately described the predictive and prognostic roles of TIL-B in BC patients.

The regulatory mechanisms of TIL-B in BC process remain largely unknown. The release of antibodies (Abs) and cytokine interleukin (IL)-10 might be involved in TIL-B-mediated tumor immunity. Furthermore, the interactions of TIL-B with other lymphocytes and the programmed death-1 (PD-1)/PD-1-ligand (PD-L1) axis (2, 3) were also well documented. Immunotherapies with agents preventing the binding of the immune checkpoint PD-1 to PD-L1 was found to be efficacious in BC (4, 5), which support a great need to fully understand the variations in TIL-B-mediated responses. Our objective is to highlight both pro- and antitumor effects of TIL-B, and underscore the regulatory mechanisms of TIL-B in the tumor environment.

## The Clinical Efficacy of TIL-B in BC

Heavy B-cell infiltrates in breast carcinoma tissues is documented and appears to be associated with a more favorable or poorer prognosis. Clinical experiments involving immunohistochemistry for analyzing the predictive and prognostic significance of TIL-B was shown in Table 1.

**Table 1:** Clinical experiments involving predictive or prognostic significance of tumor-infiltrating B cells in BC.

Year	Reference	Patients	Treatment	Surface marker	Predictive efficacy	Clinical outcomes	Independent predictive/prognosis factor
**Positively predictive or prognostic significance**							

2017	Xu et al. (15)	102 IDC	NR	CD20	NR	Improved OS and DFS (data not shown)	NR

2017	Song et al. (7)	108 primary TNBC	Post-NCT (anthracycline and taxane-based)	CD20	pCR *P* = 0.037, HR: 1.004; 95% CI: 1.000–1.007	Not with DFS (*P* = 0.194)	NR

2014	Brown et al. (6)	105 invasive BC	Post-NCT (anthracycline and taxane-based)	CD20	pCR *P* = 0.0053, OR:1.80; 95% CI: 1.19–2.72 (univariate analysis) *P* = 0.0186, OR:5.36; 95% CI: 1.32–21.8 (multivariate analysis)	NR	Independent predictive factor

2014	Garcia-Martinez et al. (8)	121 BC (stage II or III)	Post-NCT (anthracycline and taxane-based)	CD20	pCR Pre-NCT: *P* = 0.001 Post-NCT: *P* = 0.005 (OR:1.53; 95% CI: 2.2–104.1)	Not with DFS or OS (data not shown)	Independent predictive factor (for post-NCT)

2013	Mohammed et al. (13)	338 operable IDC	Adjuvant therapies (chemo-, hormonal and radiotherapy)	CD20	NR	Better BCSS (*P* < 0.01)	NR

2012	Mahmoud et al. (12)	1,470 primary invasive BC	Standard surgical of mastectomy/excision with radiotherapy	CD20	NR	Better BCSS (*P* = 0.037) Longer DFI (*P* = 0.001)	Independent prognosis factor

2010	Denkert et al. (10)	1,058 BC	Post-NCT (anthracycline and taxane-based)	CD20	pCR *P* < 0.0005	NR	NR

**Negatively prognostic significance**							

2017	Miligy et al. (24)	80 DCIS (36 pure and 44 with IDC)	Adjuvant therapies based on risk stratification	CD19, CD20, CD138	NR	Shorter RFS (*P* = 0.04)	NR

2013	Mohammed et al. (13)	338 operable IDC	Adjuvant therapies (chemotherapy, hormonal therapy, and radiotherapy)	Plasmacells; CD138	NR	Shorter RFS Plasma cells—HR: 2.96; 95% CI: 0.17–0.50, *P* < 0.001 CD138—HR:1.92, CI: 1.37–2.68, *P* < 0.001	NR

2012	Mohammed et al. (26)	468 IDC	Adjuvant therapies (chemo-, hormonal, and radiotherapy)	Plasmacells	NR	Shorter RFS (ER-negative: HR 3.25; 95% CI: 1.75–6.04, *P* < 0.001; ER-positive: HR 3.53; 95% CI: 1.97–6.29, *P* < 0.001)	Independent prognosis factor

**No predictive or prognostic significance**							

2016	Thompson et al. (35)	27 DCIS (24 pure and 3 with IDC)	NR	CD20	NR	Not with recurrence (*P* = 0.45)	NR

2012	Eiro et al. (33)	102 early IDC	Any type of neoadjuvant therapies	CD20	NR	Not with RFS (*P* = 0.956)	NR

2011	West et al. (32)	113 ER-negative invasive BC	NCT (anthracycline-based)	CD20	Fail to predict pCR (*P* > 0.1)	NR	NR

1994	Scholl et al. (29)	196 BC	NR	L26 (CD20)	NR	Not with metastases occurrence/poor survival (data not shown)	NR

*BC, breast cancer; NCT, neoadjuvant chemotherapy; pCR, pathologic complete response; BCSS, breast cancer specific survival; DFI, disease-free interval; DFS, disease-free survival; NR, not reported; ER, estrogen receptor; PR, progesterone receptor; TNBC, triple-negative breast cancer; OS, overall survival; OR, odds ratio; HR, hazard ratio; IDC, invasive ductal breast cancer; DCIS, ductal carcinoma in situ; RFS, recurrence-free survival*.

### The Positively Predictive or Prognostic Value of TIL-B in BC

#### The Positively Predictive Value

As for BC patients with neoadjuvant chemotherapy (NCT) treatment, CD20^+^ B cell infiltrates could predict pathologic complete response (pCR) (*P* = 0.005; OR, 1.80; 95% CI, 1.19–2.72) in univariate analysis. In multivariable analysis, only CD20 independently predicted pCR (*P* = 0.0186; OR, 5.368; 95% CI, 1.32–21.8), which indicated the predictive value of TIL-B through an independent manner (6). The effect was independent of age, size, nuclear grade, nodal status, estrogen receptor (ER), progesterone receptor (PR), HER2 status, and Ki67 score, which was different from other kinds of TILs. Another study also showed that in triple-negative breast cancer (TNBC) patients with NCT (7), CD20^+^ cell density was found to be a predictor of pCR (*P* = 0.037; HR, 1.004; 95% CI, 1.000–1.007). According to the aforementioned studies, posttreatment profile of high CD20^+^ B-cell infiltrates might identify a highly responsive group of BC patients. Most importantly, it supported the predictive impact of chemotherapy-mediated immune changes and identified a high-risk post-NCT tumor B-cell infiltrates condition. Moreover, TIL-B was found to predict pCR both in pre- (*P* = 0.001) and post-NCT (*P* = 0.005) settings (8), which enabled the predictive efficacy of B cells. As for the intratumoral transcript levels of TIL-B, B-cell metagenes served as an independent factor (9) which provided additional predictive information in carcinomas with high proliferative activity (HR, 0.66; 95% CI, 0.46–0.97).

In the aspect of stromal lymphocytic infiltrates, Denkert et al. (10) suggested that CD20^+^ B cells were significantly linked to pCR (*P* < 0.0005) in a total of 1,058 post-NCT BC core biopsies. Interestingly, for women with benign breast disease (11), the absence of CD20^+^ cells versus the presence in all lobules showed an adjusted OR of 5.7 (95% CI, 1.4–23.1) for subsequent cancer risk. The reduced B-cell infiltration in women with later BC suggested a role for B cells in preventing disease progression and serving as a potential biomarker for BC risk.

#### The Positively Prognostic Value

As for the prognostic effect of TIL-B (12), higher total number of CD20^+^ cell infiltrates, irrespective of location, was associated with significantly better breast cancer specific survival (BCSS) (*P* = 0.037) and longer disease-free interval (DFI) (*P* = 0.001) independently. A positive correlation was observed between higher CD20^+^ B cells number and higher tumor grade (rs = 0.20, *P* < 0.001) and ER and PR negativity (*P* < 0.001). Moreover, Mohammed et al. (13) demonstrated in invasive ductal BC patients, tumor CD20^+^ infiltrates meant a longer BCSS, while CD138^+^ B-cell exerted an opposite effect. Collectively, a current meta-analysis (14) concluded that CD20^+^ B cells were associated with better BCSS (HR, 0.77; 95% CI, 0.61–0.96) and disease-free survival (DFS) (HR, 0.72; 95% CI, 0.58–0.89). The two studies discussed above (12, 13) were included. Recently, Xu et al. (15) showed that for all types of BC patients, high CD20^+^ B cells infiltrates predicted prolonged overall survival (OS) and DFS (*P* < 0.05). However, no differences of B-cell infiltrates (16) were noted in patients with or without lymph node metastasis. Considering the limited number of patients involved (*n* = 23), future researches are expected to explore the prognostic efficacy of TIL-B in BC environment.

As for the intratumoral transcript levels of TIL-B in BC, a multivariate analysis (9) confirmed the prognostic influence of the B-cell metagene in a cohort enriched for high-grade tumors and another for younger patients. As a result, in untreated, node-negative BC patients, B-cell-related mRNA transcripts indicated a good prognosis. TNBC, a subtype with robust immune cell infiltrates, is associated with a prognostic gene signature that including B cells. Rody et al. (17) revealed that the metagene signature involving B/PC components was associated with improved survival in TNBC. As referred to above, a ratio of high B-cell and low IL-8 metagenes served as the single significant predictor to identify 32% of overall 579 TNBC patients with good prognosis (*P* < 0.001). Specifically, better prognosis was observed for high B-cell/low IL-8 group in both untreated (*P* = 0.001) and post- chemotherapy patients (*P* = 0.05). Additionally, the B-cell signature exerted predictive values in TNBC when combined with other 26-gene TNBC-derived prognostic signatures (18). In receiver operator characteristics analyses, the area under the curve (AUC) for the B-cell metagene was 0.606 (*P* = 0.025) and for the 26-gene signature was 0.588 (*P* = 0.061). A simple linear combination of both scores led to an improved AUC of 0.656 (*P* = 0.001). Furthermore, in the evaluation of prognostic potentials in NCT, Alistar et al. (19) indicated that B/PC metagenes were positively associated with distant metastasis-free survival. The protective effect, however, was mostly restricted to highly proliferative cancers of basal-like, HER2-enriched and luminal B subtypes. The results were consistent with Nagalla et al.’s research (20). All factors pointed to the predictive roles of TIL-B in BC prognosis, and emphasized the improved efficacy when combined with other TILs. However, when referred to different BC subtypes (21), the prognostic value of the B-cell/IL-8 ratio was mainly confined to the basal-like and TNBC subtypes. Recent studies utilized mRNA sequencing to analyze whether B-cell could be prognostic within specific BC subtypes. Improved metastasis-free/progression-free survival (22) was correlated with high B-cell gene signatures, which were found in basal-like and HER2-enriched subtypes.

### The Negatively Prognostic Value of TIL-B in BC

In addition to the positive function of TIL-B on antitumor reactions, compelling evidence suggests TIL-B may be associated with a worse prognosis of BC patients (shown in Table 1). In 134 cases of invasive BC and 31 breast fibroadenoma (23), the density of CD19^+^ B cells were positively associated with histological grade III, lymph node metastasis, TNM stage T4, and ER/PR negative status (indicators of poor prognosis). In particular, a significant association between CD19^+^ B-cell frequency and a small tumor size was also noticed (*P* = 0.021). To date, in tumors with mixed breast ductal carcinoma *in situ* (DCIS) (24), high density B-cell was linked to variables of poor prognosis referred to above. Outcome analysis revealed that pure DCIS with higher B-cell infiltrates presented shorter RFS (*P* = 0.04). However, no significant association was found in CD138^+^ PC count (*P* = 0.07). Other cases suggested that the predictive efficacy of PCs infiltration remains largely controversial. Of note, Parkes et al. (25) demonstrated that the presence of PCs, defined by infiltrating cells that expressed high levels of immunoglobulin (Ig) K-chain mRNA, indicated a poor prognosis. Another study also suggested (26) PCs infiltrate were related to poorer RFS, which was irrelevant to ER situation. In 338 ductal BC patients (13), tumor PCs and CD138^+^ B-cell infiltrates were both associated with poorer BCSS (*P* < 0.001) and poorer RFS (*P <* 0.001). Furthermore, another subset of BC termed PC-predominant BC (PPBC) (27) was found to indicate worse OS (HR, 2.686; *P* = 0.038) than patients with non-PPBC. All data indicated the potential prognostic efficiency of PCs infiltration, which merit future investigations.

Interestingly, TILs (28) in the metastatic lesions displayed a similar arrangement as their matched primary tumors (PT), containing CD4^+^ T cells being most abundant followed by CD8^+^ T cells and CD20^+^ B cells. Only patients with brain metastases differed by owning less CD20^+^ B cells at the infiltrative margin (*P* < 0.05). It further underlined that decreasing number of CD20^+^ B-cell might extend the aggressive behaviors of PT cancer cells metastasizing to the brain.

### No Predictive or Prognostic Value of TIL-B in BC

In 1994, Scholl et al. emphasized (29) that there was no association between L26-positive B-cell infiltration and survival. Similarly, a weak tendency of correlations was observed between CD20^+^ B cells and histological grade, Ki67 and cyclin A (30), which concluded that TIL-B did not appear to influence death in node-negative BC. In invasive ductal carcinoma tissues (31), B cells were not correlated with patients’ age, clinical, or histologic grades. No prognosis-related parameters were mentioned in the study. Another research (32) further suggested that B-cell genes failed to individually predict pCR (*P* > 0.1) in 113 ER-negative BC cases. However, they showed that anthracycline-based chemotherapy was to a certain extent more effective for CD20^high^ than for CD20^low^ patient group, suggesting a potential clinical utility of CD20^+^ TIL-B in predicting chemo-responsiveness. In post-chemotherapy residual tumor, no differences were found for B-cell infiltrates with DFS (8). In similar, Eiro et al. indicated that there was no prognostic value of CD20^+^ TIL-B alone (33), but high CD68/(CD3 plus CD20) ratio served as an independent factor in predicting shorter distant RFS (*P <* 0.01). Among TNBC patients treated with NCT (7), no significant correlation was found with DFS situation (*P* = 0.194). These findings proved that to some extent, TIL-B were not a valuable tool for predicting BC prognosis in routine pathology practice (Table 1).

As for DCIS, the frequency of CD20^+^ B cells was significantly higher in high-grade DCIS patients (34) and was associated with high-risk features. However, there was no sign of recurrence risk prediction. This was consistent with a published research by Thompson et al. (35), who found a trend for elevated numbers of CD20^+^ in ER-negative versus ER-positive DCIS cases, but CD20^+^ cells were not associated with recurrence (*P* = 0.45). Overall, there is great need to further prove the clinical utility of TIL-B and explore the correlations between TIL-B and BC prognosis.

## The Regulatory Mechanisms of TIL-B in BC

**Figure 1:**
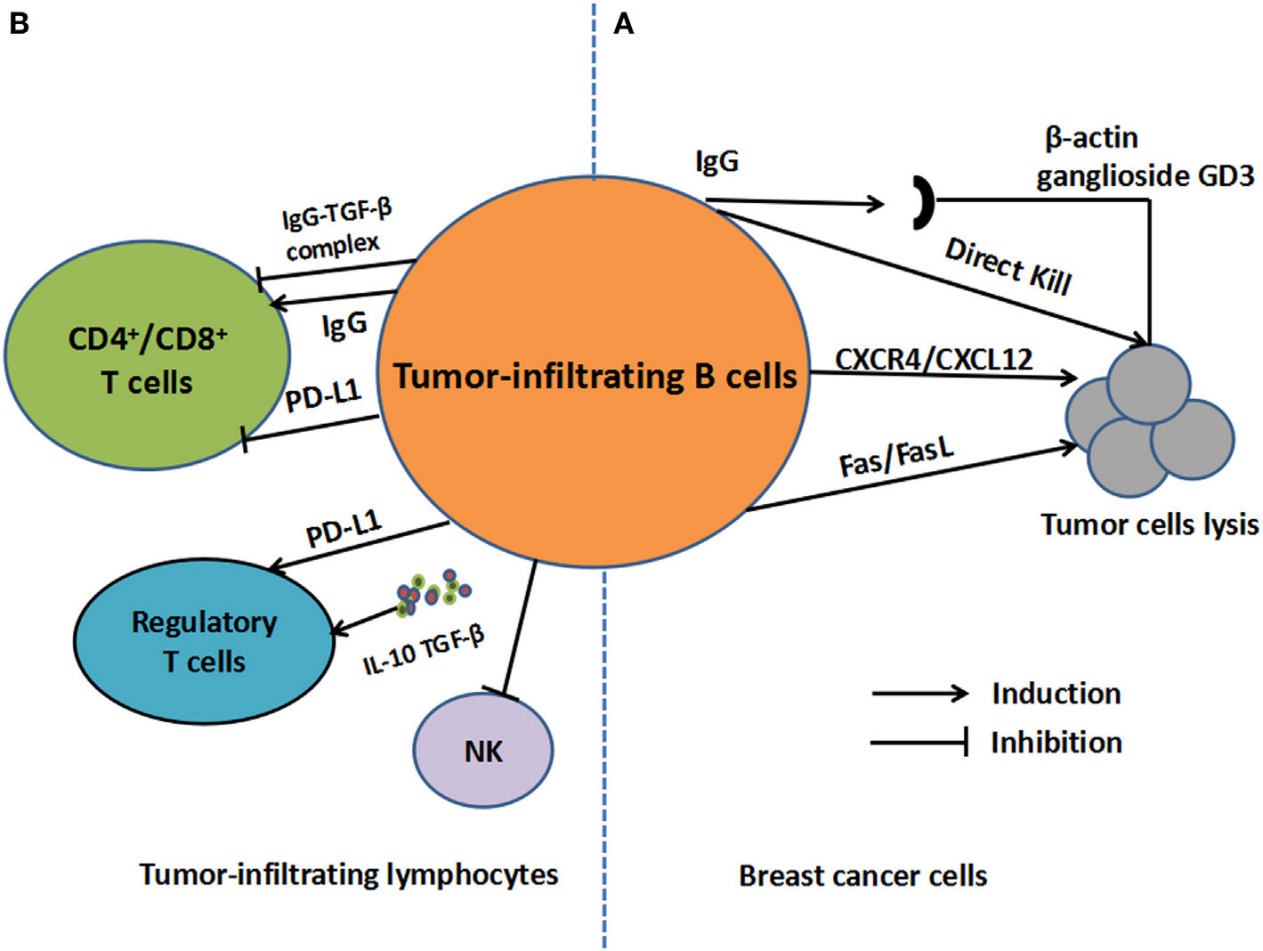
The regulatory mechanisms of tumor-infiltrating B cells (TIL-B) in breast cancer. **(A)** For tumor cells, antigens including β-actin and ganglioside GD3, which are derived from cancer cells, can be recognized by TIL-B. TIL-B can also kill tumor cells directly. Moreover, TIL-B can kill cancer cells in CXCR4/CXCL12 pathways by cell-to-cell contact or through Fas/FasL pathways. **(B)** For other tumor-infiltrating lymphocytes, regulatory functions on regulatory T cells may be engendered through secretion of IL-10 and transforming growth factor (TGF)-β as well as overexpression of PD-1-ligand (PD-L1). As for T-cell response, IgG secretion can facilitate DC-induced T-cell response or inhibit cytotoxic T cells response in the form of IgG-TGF-β complex. T cell inhibition by PD-L1 expression is also involved in the process.

Several studies focused on the aspect of TIL-B and the specific mechanisms within the overall tumor microenvironment, which are summarized in Figure 1.

### Antibodies Secretion

In the tumor microenvironment, TIL-B can secret Abs against BC antigens, which are potent mechanisms of tumor control. To be more specific, antigens mainly including β-actin and ganglioside GD3, which were derived from cancer cells, could be recognized by TIL-B (36–38). In medullary carcinomas (MC), TIL-B-derived auto-Abs were shown to recognize these antigens; hence, directed against an intracellular protein trans-located to the cell surface upon MC cell apoptosis. The apoptotic MC cells were shown to expose actin on cell surface, permitting its recognition by TIL-B-secreting auto-Abs (37). More specific, the TIL-B (36) in half of the MC patients mainly exhibited an IgG2 response, which was an antigen-derived early apoptotic event. The process can facilitate the translocation and proteolytic fragmentation of intracellular proteins. Another study (38) showed that the capacity of TIL-B was to reveal key cancer-related antigens, such as the GD3 ganglioside, which influenced tumor cell progression. In addition, IgG subtype secretion (39) can also facilitate DC-induced T-cell response; hence, prevent BC relapse (40). Activated 4T1 tumor-draining lymph node (TDLN) B cells secreted IgG in response to tumor cells through an immunologically specific manner. These activated B cells were capable of mediating specific lysis of tumor cells *in vitro*. Transfer of activated B cells alone inhibited spontaneous metastases of cancer cells to the lung. The transfer also resulted in the induction of tumor-specific T-cell immunity as measured by cytotoxicity and the productions of cytokine interferon (IFN)-γ and granulocyte-macrophage colony-stimulating factor. In similar, TDLN B cells plus IL-2 administration (41) produced larger amounts of IgG, binding to 4T1, and led to cancer cells lysis. The study also suggested that TDLN B cells could kill cancer cells in CXCR4/CXCL12 pathways by cell-to-cell contact, which termed to be an additive manner. The CXCR4 expression on B cells lead to cytotoxicity of CXCL12- producing tumor cells. Without cell contact, perforin also play a role in B cell-mediated tumor cell cytotoxicity in TDLN (41).

Antibodies binding to tumor antigens may induce powerful antitumor response, underscoring the essential roles of TIL-B in the BC environment. The production of Abs binding to specific tumor-associated antigens (TAA) on BC cells can trigger natural killer (NK) cells to bind to the Fc portion of Abs, resulting in tumor cell lysis through antibody-dependent cell-mediated cytotoxicity (ADCC). In BC environment, ADCC is a vital immune mechanism sculpted by evolution to eradicate tumor cells, in which tumor cells can be recognized by tumor antigen-specific Abs. For example, a typical TAA termed polymorphic epithelial mucin (MUC1) was observed to play a role in BC immunity. Fremd et al. observed that high anti-MUC1 IgG levels were positively associated with improved OS in BC patients (42). A previous study (43) also concluded that in early stage BC, both serum MUC1 IgG and IgM Abs were associated with a significant benefit in disease-specific survival. The MUC1 Abs might further aid in the control of tumor dissemination and identify better clinical outcomes of BC patients. Currently, Gheybi et al. (44) proved that the recombinant HER2-MUC1 as a chimeric protein vaccination in a mouse model to develop a more efficacious vaccine against BC. These findings implied a critical role of MUC1 as a vital TAA in B-cell-mediated tumor immunity. In conclusion, a tumor antigen-specific immune response triggered by TIL-B may contribute to the regulations of BC immunity.

### IL-10 Production

Within the tumor environment, regulatory B cells (Bregs) are widely recognized to inhibit immune responses through IL-10 production (45). However, the inner mechanisms of IL-10 secretion by TIL-B and the roles in breast tumor sites remained to be elaborately concluded. A typical type of B220^+^CD25^hi^CD69^hi^MHC-II^hi^ B-cell subset (46) could promote lung metastases of BC, which secrete cytokine transforming growth factor (TGF)-β and IL-10. The suppressive effect of B cells in the tumor setting was partly relied on IL-10 secretion. However, the primary role of TIL-B was to induce the conversion of CD4^+^ T cells to FoxP3^+^ regulatory T cells (Tregs) in a TGF-β-dependent manner. Additionally, Tao et al. (47) demonstrated IL-10-producing B cells derived from TDLN could kill BC cells through Fas/FasL pathways. IL-10 is an essential regulator in the process. The absence of IL-10 led to augmented therapeutic efficacy of adoptively transferred TDLN B cells. However, in EMT-6 murine models, mice that are genetically lacking in B cells (BCDM) showed a reduced tumor growth. The adopt transfer of wide type or IL-10^−/−^ B cells both restored tumor growth and reduced survival relative to BCDM (48, 49), which suggested that IL-10 secretion may be not necessary in restoring tumor growth in murine models. As a result, the production of IL-10 might be partly involved in the regulatory mechanisms of B-cell infiltrates in PT or TDLN, which deserved future investigations.

### The Regulatory Effects on Other Lymphocytes

Within the tumor environment, purified TIL-B (50) were reported to suppress the proliferation of CD4^+^, CD8^+^, and CD4^+^CD25^−^ T cells, as well as NK proliferation in response to IL-15. Extensive B-cell infiltration demonstrated impaired cytotoxic T cells (CTL) response when compared with that in B-cell-deficient μ^(−/−)^ mice. In EMT-6 murine models, tumors show reduced growth in BCDM. The B-cell deficiency (49) was accompanied by increased T and NK cell infiltration, a more vigorous Th1 cytokine response, and an increased CTL response. In addition, a decreased number of CD4^+^CD25^+^FoxP3^+^ Tregs was noted in the spleen of BCDM and TDLN relative to wide-type mice. Consistent with these results, Zhang et al. demonstrated that high frequencies of intratumoral B cells were associated with increased recruitment and proliferation of Tregs (48). The accumulations of the two cells might contribute to reduced infiltrations of NK and CD8^+^T cell in the tumor sites, resulting in an impaired T-cell response. Another typical Breg infiltrates (46) was able to induce the conversion of CD4^+^ T cells to FoxP3^+^ Tregs in a TGF-β-dependent manner, which promoted lung metastases of BC. A previous study also proved B lymphocytes secreting IgG linked to latent TGF-β to prevent CTL responses (51). However, a research demonstrated that the adoptive transfer of TDLN-derived B cells led to the enhanced tumor-specific T-cell response (40) with increasing productions of IFN-γ and GM-CSF. It underscored the vital effect of TIL-B on T cell-mediated immunity, which further resulted in the inhibition of 4T1 spontaneous pulmonary metastases. A atypical Granzyme B (GrB)-secreting B cells (52) termed CD19^+^ CD20^+^ CD27^−^ CD38^−^ IgD^−^ can upregulate several molecules interacting with other immune cells, including costimulatory, antigen-presenting, and the cell adhesion molecules, hence, might exert regulatory effects on other TILs. In addition, in BC patients, GrB (53) was highly expressed in one-third of the chemotherapy-treated tumors, and suggested an excessive CTL response within tumors after exposure to chemotherapy. The presence of GrB-secreting TIL-B might contribute to positively immune response in the tumor environment. The findings aforementioned indicated the collaborative interactions between TIL-B with other lymphocytes, which provided alternative strategies in developing more effective cellular therapies.

### The Interactions with PD-1/PD-L1 Axis

Several studies have identified PD-L1 as a critical mediator of Bregs. Guan et al. (23) reported that PD-L1 contributed to the immunosuppressive role of CD19^+^CD24^+^CD38^+^ Bregs in invasive BC patients. PD-L1 expression on B cells can result in the inhibition of T-cell proliferation (54) and T-cell-dependent immunogenic chemotherapy (51). The PD-L1 expression on B cells contributes to the B-cell suppressive properties, which may act as therapeutic targets for BC treatment.

Recent studies have indicated that an increased TILs presence at the tumor site is linked with the efficacy of immune checkpoint blockade (55). PD-L1 expression in BC was proved to be associated with poor prognosis (56). Clinical utility of immune checkpoint inhibitors (ICIs) has exerted long-lasting responses and survival benefits (57). However, their specific correlations with TIL-B and the acting mechanisms remain unclear. Patients with high B-cell infiltrates might benefit from ICIs treatment, and the underlying mechanisms are described as follows. Buisseret et al. suggested (58) that B-cell infiltrates in tumor tissue might be induced by immunotherapies targeting PD-1/PD-L1 axis. Specifically, PD-L1 expression was associated with higher TILs density including CD19^+^ B cells. TILs density, tertiary lymphoid structure, and PD-L1 expression were correlated with more aggressive tumor characteristics. These data emphasized the relationship between PD-1/PD-L1 expression and increased B-cell infiltration which represents a potential approach for antitumor treatment. Another similar study (23) observed that TIL-B were highly coincident with PD-L1 and IL-10 in invasive BC. Moreover, a typical subgroup of TIL-B termed CD19^+^CD24^+^CD38^+^ B cells can induce the formation of Tregs when cocultured with T cells from BC patients and healthy subjects (80.4 and 30.8%, respectively). The regulatory process was proved to be mediated by PD-L1. In 4T1 murine BC models (50), PD-L1 expression was significantly increased in TIL-B relative to splenic B cells, which demonstrated enhanced inhibitory activity against CD4^+^/CD8^+^ T cells and NK cells. It underlies the potential interactions between PD-L1 expression and inhibitory abilities of TIL-B. Together, these results were suggestive of a role for CD19^+^ B lymphocytes in immune suppression and tumor evasion *via* PD-L1 in BC.

## Conclusion

Current evidence suggests that the B-cell infiltrates in BC tissues might perform either positive, negative, or none significant in humoral and cellular immunity of BC. Abs and IL-10-secreting B cells are involved in the process. Moreover, there are potential inter-connections between TIL-B with other lymphocytes and the efficacy of ICIs (Figure 1). Therapies targeting B-cell might contribute to an improvement in treatment efficacy. Additionally, other B cell-related mechanisms, such as the transcriptional regulations of TIL-B, can also be a promising option to affect clinical outcomes. Blimp1 is a transcriptional repressor that controls gene expression changes in B-cell-to-plasmablast transition, which led to silences of B-cell gene expression in PC (59). High Blimp1 expression tends to trigger BC cell invasion and metastasis formation (60). Thus, targeting B-cell transcriptional regulators may further influence BC progression and invasiveness, which reinforces the potential of B cells as a promising therapeutic target. However, currently, neither histopathological features nor conventional biomarkers can be accurately and commonly recognized. The understanding of regulatory mechanisms will aid in predicting clinical outcomes of BC patients and emerge as a guide to determine the more effective therapies.

## Author Contributions

XR designed the review. MS and XR drafted the manuscript and finalized the table and figure. JW provided useful suggestions. All authors read and approved final manuscript.

## Conflict of Interest Statement

The authors declare that the research was conducted in the absence of any commercial or financial relationships that could be construed as a potential conflict of interest.

## Abbreviations

BC, breast cancer; OS, overall survival; TILs, tumor-infiltrating lymphocytes; TIL-B, tumor-infiltrating B cells; Bregs, regulatory B cells; Tregs, regulatory T cells; PD-1, programmed death-1; PD-L1, PD-1-ligand; pCR, pathologic complete response; NCT, neoadjuvant chemotherapy; BCSS, breast cancer specific survival; DFI, disease-free interval; RFS, recurrence-free survival; DFS, disease-free survival; TNBC, triple-negative breast cancer; MC, medullary carcinomas; PFS, progression-free survival; DCIS, ductal carcinoma *in situ*; mTIL, metastasis infiltrating lymphocytes; PT, primary tumors; TLS, tertiary lymphoid structure; ICIs, immune checkpoint inhibitors; ER, estrogen receptor; PR, progesterone receptor; CTL, cytotoxic T cells; ROC, receiver operator characteristics; GrB, Granzyme B; Ab, antibody; AUC, area under the curve; TAA, tumor-associated antigens; ADCC, antibody-dependent cell-mediated cytotoxicity; MUC1, polymorphic epithelial mucin.
